# Ensemble Species Distribution Modeling of Climate Change Impacts on Endangered Amphibians and Reptiles in South Korea

**DOI:** 10.3390/ani16010095

**Published:** 2025-12-29

**Authors:** Jae-Ho Lee, Min-Ho Chang, Man-Seok Shin, Eun-Seo Lee, Jae-Seok Lee, Chang-Wan Seo

**Affiliations:** 1 National Institute of Ecology, Seocheon 33657, Republic of Korea; jhlee14@nie.re.kr (J.-H.L.); mhchang@nie.re.kr (M.-H.C.); manhae@nie.re.kr (M.-S.S.); pooky84@nie.re.kr (E.-S.L.); 2 Department of Biological Sciences, Konkuk University, Seoul 05029, Republic of Korea; jaeseok@konkuk.ac.kr

**Keywords:** species distribution modeling, ensemble modeling, climate change, endangered species, amphibians, reptiles, niche breadth, SSP scenarios, South Korea

## Abstract

Climate change poses a serious threat to amphibians and reptiles, which are especially vulnerable because of limited thermoregulatory capacity and restricted dispersal. We used an ensemble species distribution modeling framework to assess habitat determinants, niche breadth, and climate-driven distribution changes for eight legally protected endangered amphibian and reptile species in South Korea. Occurrence records collected between 1997 and 2021 were combined with ten bioclimatic, topographic, and hydrological predictors, and 11 species distribution modeling algorithms (SDMs), including Random Forest and MaxEnt, were implemented and combined into weighted ensemble predictions. The weighted ensemble model showed high predictive performance (mean ROC–AUC = 0.897; overall mean across all SDMs = 0.843). Variable-importance analysis revealed clear taxonomic contrasts: reptiles exhibited approximately 1.7-fold greater dependence on temperature variables than amphibians, whereas amphibians were more strongly associated with precipitation and topographic context. Environmental niche-breadth analysis identified *Sibynophis chinensis*, *Hynobius yangi*, and *Dryophytes suweonensis* as narrow- or moderate-niche specialists largely constrained by precipitation of the driest month and a small set of climatic variables. Under moderate (SSP2-4.5) and high (SSP5-8.5) emission scenarios, areas of high species richness are projected to decline by 22% and 45%, respectively, by the 2070s, with distribution centroids shifting northeastward and pronounced habitat loss in western lowland plains. Priority conservation targets include *S. chinensis*, *D. suweonensis*, and *H. yangi*, which combine narrow niches, restricted ranges, and high climate vulnerability. These findings provide a quantitative basis for climate-adaptive conservation planning for threatened herpetofauna in South Korea.

## Simple Summary

Climate change and habitat loss threaten amphibians and reptiles worldwide, with many species facing extinction. In South Korea, eight amphibian and reptile species are officially designated as endangered, but we lack detailed information about their habitat requirements. This study used computer modeling to predict suitable habitats for these species. We found that each species has unique environmental needs: amphibians depend on precipitation patterns, especially dry season rainfall, whereas reptiles are more affected by temperature. Some species are habitat specialists with narrow requirements, making them vulnerable to environmental changes, while others are generalists adapting to various conditions. Using climate change projections, we predicted that suitable habitats will dramatically shrink by the 2070s, particularly in western lowland plains that currently support highest diversity. Northern mountain regions may become future refuges as the climate changes, but many species may not be able to reach these areas on their own because their dispersal is limited and habitats are fragmented. Under high emission scenarios, suitable habitat areas could decline by forty-five percent, but moderate emission pathways could reduce this loss by half. Our findings provide essential information for conservation planning, identifying which species need urgent protection and where conservation efforts should focus to prevent extinctions.

## 1. Introduction

Climate change poses severe threats to global biodiversity, and ectothermic vertebrates are among the most vulnerable groups due to constrained thermoregulation and limited dispersal capacity [1,2]. Amphibians often show high sensitivity to coupled temperature–moisture changes because of permeable skin and complex life histories [3,4], while reptiles rely strongly on ambient thermal regimes that regulate activity, growth, and reproduction [5,6]. As a result, amphibians and reptiles exhibit high extinction risk globally, and their vulnerability is further amplified by the combined effects of climate change and ongoing habitat loss [7,8].

South Korea is characterized by strong seasonality and substantial precipitation variability, and rapid land-use change has intensified pressures on freshwater- and wetland-associated habitats as well as lowland landscapes [9]. These changes threaten endangered amphibians and reptiles through habitat degradation, fragmentation, and reduced connectivity between breeding and foraging sites [10,11]. Although national monitoring programs have accumulated substantial occurrence information, quantitative synthesis of habitat requirements and climate-change responses remains limited for many taxa, which constrains proactive conservation planning [12]. Identifying the environmental factors that shape current distributions and anticipating future shifts in climatic suitability are therefore essential for climate-adaptive management and prioritization [13,14].

Species distribution models (SDMs) provide a practical approach for linking species occurrences with environmental predictors and projecting potential habitat suitability in space and time [15,16]. However, single-algorithm SDMs can be sensitive to modeling assumptions and sampling artefacts; ensemble frameworks that integrate multiple algorithms can reduce uncertainty and improve robustness by combining complementary model strengths [17,18]. In addition, quantifying niche breadth based on predictor-contribution profiles can help distinguish species with narrow, concentrated environmental dependence from those with broader multi-factor dependence, which is useful for anticipating climate vulnerability and tailoring conservation actions [19,20]. Multivariate comparisons of predictor-contribution patterns can also reveal ecological similarity among species and inform coordinated conservation strategies when multiple taxa respond to shared limiting factors [21,22]. Beyond predictive mapping, SDMs are increasingly used to support systematic conservation planning and conservation priority setting under environmental change [23,24]. This is particularly relevant given the documented consequences of biodiversity loss for ecosystem functioning and human well-being [25]. Such SDM-based outputs can help guide conservation decisions by identifying areas expected to retain suitability under future climates [26].

Here, we evaluate climate-sensitive habitat suitability for eight endangered amphibian and reptile species in South Korea using an ensemble SDM framework. We combine national-scale occurrence datasets with high-resolution climatic and environmental predictors to (i) identify key environmental drivers and map current habitat suitability, (ii) quantify niche breadth and similarity in modeled environmental constraints, and (iii) project future habitat suitability and species richness under moderate and high-emission trajectories. Shared Socioeconomic Pathways (SSPs) provide standardized scenario frameworks for assessing biodiversity responses to plausible future forcing pathways [27,28], enabling projections under contrasting levels of warming and hydroclimatic change [29]. Because richness patterns summarize multi-species responses and can indicate potential refugial areas, we also evaluate spatial shifts in richness and highlight areas likely to retain suitability for multiple taxa [30,31]. Together, these analyses provide an evidence base to support climate-adaptive conservation planning and prioritization in South Korea.

## 2. Materials and Methods

### 2.1. Study Area

**Figure 1:**
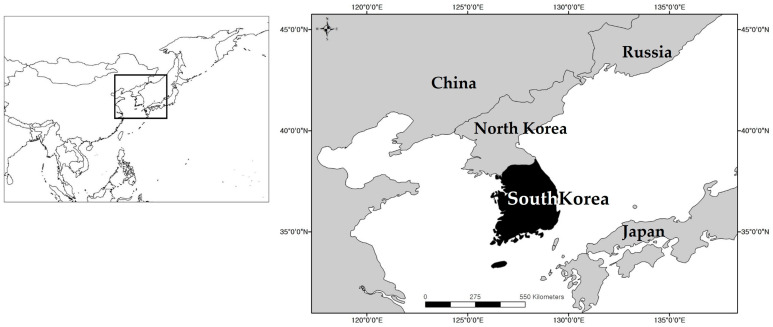
Location of the study area in South Korea. The inset map shows the position of the Korean Peninsula within East Asia, and the main map highlights South Korea (black) with neighboring countries labeled for geographic context.

This study was conducted across mainland South Korea, which occupies the southern Korean Peninsula in Northeast Asia (area ≈ 100,000 km^2^; Figure 1). The country has a temperate monsoon climate, with mean annual temperature of approximately 12–14 °C and mean annual precipitation of 1200–1500 mm for the 1991–2020 normal period; about 60–70% of annual rainfall occurs during the June–August summer monsoon [9]. Floristic analyses further subdivide South Korea into cool-temperate, cool-temperate montane, warm-temperate, and oceanic warm-temperate regions. Topographically, the Baekdudaegan mountain range forms a north–south spine of steep highlands in the east, whereas low hills and broad alluvial plains dominate the west and south [32]. This interaction of climate and topography creates a mosaic of rivers, agricultural lowland wetlands, inland marshes, and forested mountain valleys that provides diverse habitats for amphibians and reptiles. For species distribution modeling, the entire country was partitioned into 1 km × 1 km grid cells, and environmental conditions and habitat suitability were evaluated at the grid-cell level.

### 2.2. Target Species

We selected eight threatened amphibian and reptile species that are legally protected in South Korea and represent a range of ecological traits and habitat requirements (Table 1). According to the National Species List of Korea compiled by the National Institute of Biological Resources (NIBR; https://species.nibr.go.kr/, accessed on 16 August 2025), three species are classified as Endangered (EN)—*Hynobius yangi*, *Dryophytes suweonensis*, and *Sibynophis chinensis*—whereas five species are listed as Vulnerable (VU)—*Pelophylax chosenicus*, *Kaloula borealis*, *Eremias argus*, *Elaphe schrenckii*, and *Mauremys reevesii*. The national legal protection status further distinguishes conservation priority among these taxa. *D. suweonensis* and *S. chinensis* are designated as Endangered Wildlife Class I by the Korean Ministry of Environment, reflecting their particularly high risk of extinction and the need for strict protection. The remaining six species—*H. yangi*, *P. chosenicus*, *K. borealis*, *E. argus*, *E. schrenckii*, and *M. reevesii*—are classified as Endangered Wildlife Class II. Overall, the target set consists of four amphibians (*H. yangi*, *D. suweonensis*, *P. chosenicus*, *K. borealis*) and four reptiles (*S. chinensis*, *E. argus*, *E. schrenckii*, *M. reevesii*), providing a balanced representation of endangered herpetofauna for assessing climate-related habitat changes and conservation priorities [33].

### 2.3. Species Occurrence Data

Occurrence records for eight nationally protected amphibian and reptile species were compiled from three government-led monitoring programs: (i) the Third National Ecosystem Survey (1997–2022) conducted by the National Institute of Ecology, (ii) Korea National Park Service monitoring (2003–2022) to improve coverage of protected mountainous areas, and (iii) long-term monitoring records from the Endangered Species Restoration Center for known populations. All records included geographic coordinates (WGS84 datum) with spatial accuracy <1 km [34]. Because these datasets originate from standardized national surveys and protected-area monitoring, species identifications were made by trained observers following official survey protocols; records with ambiguous identification or insufficient location information were excluded during data curation.

To minimize spatial autocorrelation and sampling bias, spatial thinning was applied by retaining only one occurrence record per 1 km × 1 km grid cell, matching the resolution of the environmental predictors. Distance-based weighting was applied to records concentrated along roads and trails to partially account for accessibility bias. Species-specific sample sizes before and after 1 km thinning are summarized in Table 2. These national-scale datasets collectively provide broad geographic coverage across mainland South Korea, spanning major physiographic regions and including both protected and non-protected areas. Nevertheless, as is common in biodiversity occurrence data, sampling intensity can remain spatially heterogeneous and may be higher in more accessible areas (e.g., near roads and trails). We partially addressed this issue through 1 km spatial thinning and distance-based weighting, but some residual spatial gaps and temporal clustering may persist and should be considered when interpreting model projections.

### 2.4. Environmental Variables

Species distribution models were parameterized using 10 environmental predictors representing climatic, topographic, and hydrological constraints relevant to amphibians and reptiles (Table 3). Climatic predictors comprised six bioclimatic variables (BIO1, BIO2, BIO3, BIO12, BIO13, BIO14) derived from high-resolution climate data and standard bioclimatic formulations [35,36]. Topographic predictors included elevation, slope, and the topographic wetness index (TWI) derived from a digital elevation model using standard terrain-processing workflows [37]. Hydrological accessibility was represented by distance to water. All predictor layers were harmonized to a common 1 km × 1 km grid, projection, and spatial extent covering mainland South Korea.

To assess multicollinearity, we calculated pairwise Pearson correlation coefficients among predictors across the study area and treated |r| > 0.7 as indicative of strong collinearity [38]. In our predictor set, the only pair exceeding this threshold was BIO2 (mean diurnal range) and BIO3 (isothermality; Pearson’s r = 0.715). Because strong collinearity was limited to a single, conceptually related pair and all predictors have clear ecological relevance, we retained the full set of 10 predictors for model fitting and interpreted variable contributions as relative importance measures rather than mechanistic effects.

Topographic variables were derived from a digital elevation model, and TWI was computed using standard terrain-based procedures. Distance to water was calculated as Euclidean distance to the nearest mapped stream or water body. Predictor selection was guided by established SDM practice and by the known climatic and habitat sensitivities of herpetofauna, particularly temperature and precipitation seasonality and proximity to aquatic environments.

**Table 3:** Environmental variables used in species distribution modeling.

Category	Variable Name	Code	Unit	Source
Bioclimatic	Annual Mean Temperature	BIO1	°C	KMA
Bioclimatic	Mean Diurnal Range	BIO2	°C	KMA
Bioclimatic	Isothermality	BIO3	%	KMA
Bioclimatic	Annual Precipitation	BIO12	mm	KMA
Bioclimatic	Precipitation of Wettest Month	BIO13	mm	KMA
Bioclimatic	Precipitation of Driest Month	BIO14	mm	KMA
Topographic	Elevation	elevation	m	National Geographic Information Institute
Topographic	Slope	slope	degrees	National Geographic Information Institute
Topographic	Topographic Wetness Index	wetness	-	Calculated from DEM
Hydrological	Distance to Water	dist_water	km	Ministry of Environment (WAMIS)

Note: Bioclimatic variables were obtained from KMA [39]. Topographic variables (slope, curvature, and TWI) were derived from the NGII DEM [40]. Distance to river was calculated using the river network from MOE WAMIS [41].

### 2.5. Species Distribution Modeling

#### 2.5.1. Modeling Approaches

Species distribution modeling was conducted in R 4.3.0 using the biomod2 package (version 4.3-4-3) [38]. We implemented 11 modeling approaches comprising nine single-algorithm species distribution models and two ensemble models. The single-algorithm models were: Random Forest (RF), Gradient Boosting Machine (GBM), Generalized Linear Model (GLM), Flexible Discriminant Analysis (FDA), Multivariate Adaptive Regression Splines (MARS), Artificial Neural Network (ANN), Maximum Entropy (MaxEnt), Classification Tree Analysis (CTA), and Surface Range Envelope (SRE) [42,43,44,45]. Ensemble model construction is described in Section 2.5.4.

#### 2.5.2. Model Calibration and Validation

Model performance was evaluated using 5-fold cross-validation. Occurrence data were randomly partitioned into five folds (80% training, 20% validation) and repeated across 10 independent runs per algorithm, yielding 50 models per species for each algorithm. Performance was assessed using the True Skill Statistic (TSS) [46], ROC-AUC [47], Cohen’s Kappa [48], sensitivity, and specificity. Binary predictions were derived using the threshold that maximized TSS. In the absence of true absence data, pseudo-absence points were generated for presence–pseudo-absence algorithms in biomod2 using a random sampling strategy (PA.strategy = “random”). For each species, 10,000 pseudo-absences (PA.nb.absences = 10,000) were sampled across mainland South Korea from grid cells with complete predictor coverage, excluding 1 km × 1 km cells containing presence records. Pseudo-absence sampling was replicated 10 times (PA.nb.rep = 10) to account for uncertainty in pseudo-absence placement, and identical settings were applied across algorithms to ensure comparable evaluation. Random cross-validation can overestimate performance when spatial autocorrelation is present. We reduced spatial clustering using 1 km thinning and accessibility-aware weighting (Section 2.3), but residual spatial dependence may remain. After thinning, some species had small sample sizes (e.g., *n* = 23–25), limiting the feasibility of spatial block cross-validation; therefore, performance metrics and projections should be interpreted cautiously.

#### 2.5.3. Variable Importance Analysis

Relative contribution of each environmental variable was assessed using permutation-based variable importance [43]. This approach quantifies variable importance by measuring model performance reduction after randomly permuting variable values. Variable contributions were normalized per species to sum to 1.0, enabling direct interspecific comparisons.

#### 2.5.4. Ensemble Modeling

We generated ensemble predictions to reduce dependence on any single algorithm and to summarize consensus suitability across models. Four ensemble strategies available in biomod2 were evaluated: ensemble mean, ensemble median, committee averaging, and AUC-weighted mean [49]. For committee averaging, each single-model prediction was converted to binary presence/absence using the maxTSS threshold, and the ensemble value represents the proportion of models predicting presence. For the AUC-weighted mean, continuous suitability predictions were combined using model weights proportional to discriminatory performance (ROC-AUC), with weights rescaled to sum to 1 (after baseline adjustment so that poorly performing models receive negligible weight). Because AUC-weighted mean performed best overall and provided stable spatial patterns, it was used as the primary ensemble output for mapping and subsequent analyses, while the other ensemble strategies were used for sensitivity checking.

### 2.6. Habitat Suitability and Future Projections

#### 2.6.1. Current Habitat Suitability Mapping

Ensemble model outputs generated continuous habitat suitability values ranging from 0 to 1. Binary classification thresholds were determined by maximizing TSS, with values exceeding the threshold classified as suitable habitat [50]. Habitat suitability maps were produced at 1 km × 1 km resolution using the raster and terra packages in R 4.3.0 [51] with WGS84 coordinate system.

#### 2.6.2. Future Climate Scenarios

Future climate change impacts were projected under two IPCC AR6 SSP scenarios: SSP2-4.5 (moderate emission pathway) and SSP5-8.5 (high emission pathway) [27,28]. SSP2-4.5 represents continuation of current climate policies with radiative forcing reaching 4.5 W/m^2^ by end-century. SSP5-8.5 represents fossil fuel-intensive development reaching 8.5 W/m^2^ radiative forcing [29]. Future climate data at 1 km resolution were obtained from KMA, generated through statistical ensembling of five regional climate models (HadGEM3-RA, WRF, CCLM, GRIMs, RegCM4) [39]. Projections were evaluated for three time periods: 2030s (2021–2040), 2050s (2041–2060), and 2070s (2061–2080), each representing 20-year averages.

#### 2.6.3. Model Projection and Species Richness Analysis

Ensemble models calibrated on current climate (1981–2010 normals) were projected to future climate conditions. Topographic variables (elevation, slope, TWI) and distance to water were assumed temporally static [52]. Model projections employed clamping to minimize extrapolation errors when predicted environmental values exceeded training data ranges [53]. Species richness was calculated as the sum of species predicted present (suitability exceeding the maxTSS threshold) per 1 km^2^ grid cell [30]. For each species, changes in suitable area (%) were calculated as 100 × (Afuture − Acurrent)/Acurrent, where Acurrent and Afuture denote the total number of suitable 1-km^2^ grid cells under current and future conditions, respectively. Species richness maps were generated for current conditions and each future scenario–period combination, and temporal change patterns were quantified accordingly.

### 2.7. Statistical Analysis

Variable contributions were normalized per species to sum to 1.000, enabling direct comparisons independent of model performance or sample size. Niche breadth was quantified using the Shannon diversity index calculated from the normalized variable-contribution profile for each species [54,55]. With S = 10 predictors, the theoretical maximum is H′max = ln(10) = 2.303. We used H′ as a continuous measure and additionally applied a descriptive categorization to facilitate interpretation: specialist (H′ < 1.60), moderate specialist (1.60 ≤ H′ ≤ 1.95), and generalist (H′ > 1.95). These cutoffs were set to separate lower, intermediate, and higher fractions of H′max and are intended as descriptive groupings rather than universal ecological thresholds.

Taxonomic differences (amphibians vs. reptiles) in variable contributions were tested using Welch’s two-sample *t*-test [56]. Model performance metrics (TSS, ROC-AUC) were compared between taxonomic groups using the Mann–Whitney U test [57]. Because sample size per group was small (*n* = 4), these comparisons were treated as exploratory and interpreted alongside effect sizes (Hedges’ g for *t*-tests and rank-biserial correlation for Mann–Whitney tests) and uncertainty estimates. Outliers in variable-contribution patterns were screened using Grubbs’ test (α = 0.05) [58]. Interspecific similarity in modeled predictor-contribution profiles was assessed using Spearman rank correlation coefficients. Variable intercorrelation was evaluated using Pearson correlation coefficients across the eight species’ variable contributions. Hierarchical cluster analysis employed Ward’s minimum variance method [59] with distance defined as 1 − Spearman’s ρ. Dendrogram fit was assessed using the cophenetic correlation coefficient, with values ≥0.8 considered adequate representation of the original distance matrix [60]. All statistical analyses were performed in R (version 4.5.2) at a significance level of α = 0.05. Shannon index calculations were conducted using the vegan package (version 2.7-2) [61], and hierarchical clustering was performed with the hclust function in the stats package (base R).

Importantly, clustering summarizes similarity in modeled predictor-contribution profiles (i.e., which variables most influenced suitability) and should not be interpreted as evidence of true niche overlap, co-occurrence, or species interactions. Accordingly, clustering results are treated as exploratory and are used to generate hypotheses for follow-up validation.

## 3. Results

### 3.1. Model Performance and Validation

#### 3.1.1. Overall Model Accuracy

**Figure 2:**
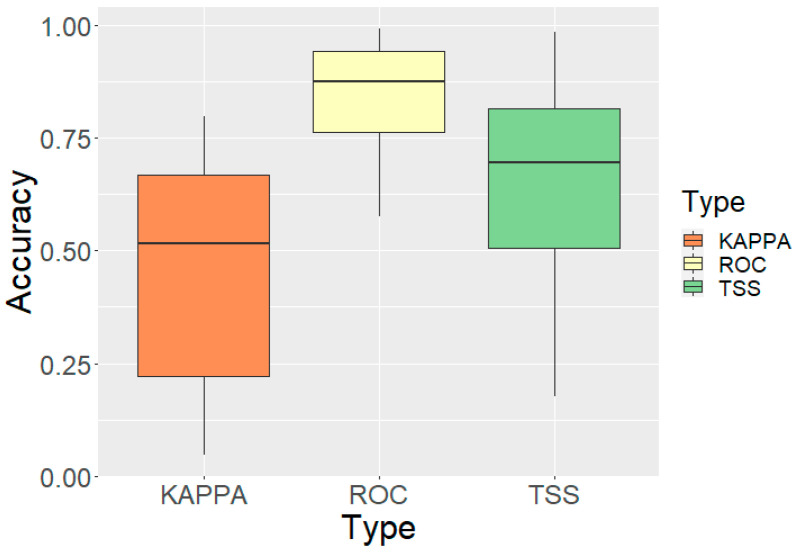
Model performance metrics (ROC-AUC, TSS, and Cohen’s Kappa) across validation runs for single algorithms and ensemble approaches.

The ensemble species distribution models demonstrated robust predictive performance across all eight endangered amphibian and reptile species. Mean ROC-AUC across all species and models was 0.843 ± 0.120 (Table 4, Figure 2). The True Skill Statistic (TSS) yielded a mean value of 0.654 ± 0.221. Cohen’s Kappa averaged 0.460 ± 0.231. Wide ranges were observed in all metrics (ROC-AUC: 0.575–0.992; TSS: 0.177–0.985; Kappa: 0.047–0.797), showing substantial interspecific variation in model predictability.

#### 3.1.2. Model Performance Comparison

**Figure 3:**
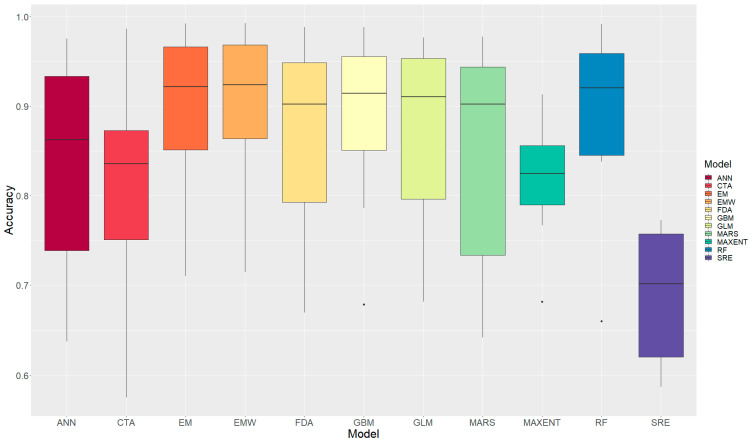
Accuracies of all models. Black dots indicate the minimum value (i.e., the lowest score) for each model.

Ensemble modeling approaches consistently outperformed individual models (Table 5, Figure 3). The ensemble weighted mean (EMW) achieved the highest performance with a mean ROC-AUC of 0.897 ± 0.099, followed closely by the ensemble mean (EM; 0.892 ± 0.103) and Random Forest (RF; 0.889 ± 0.108). Among machine learning models, Gradient Boosting Machine (GBM; 0.885 ± 0.105) and Generalized Linear Model (GLM; 0.864 ± 0.118) ranked fourth and fifth, respectively. Surface Range Envelope (SRE) exhibited the poorest performance (0.690 ± 0.074). Algorithm stability, assessed through coefficient of variation (CV), is shown in Table 6. Maximum Entropy (MAXENT) demonstrated the lowest variability across species (CV = 8.7%). The ensemble approaches (EMW: 11.0%; EM: 11.5%) and machine learning methods (GBM: 11.9%; RF: 12.1%) showed moderate stability. In contrast, Classification Tree Analysis (CTA; 17.0%), Artificial Neural Network (ANN; 16.3%), and Multivariate Adaptive Regression Splines (MARS; 15.9%) exhibited high variability.

#### 3.1.3. Species-Specific Model Performance

**Figure 4:**
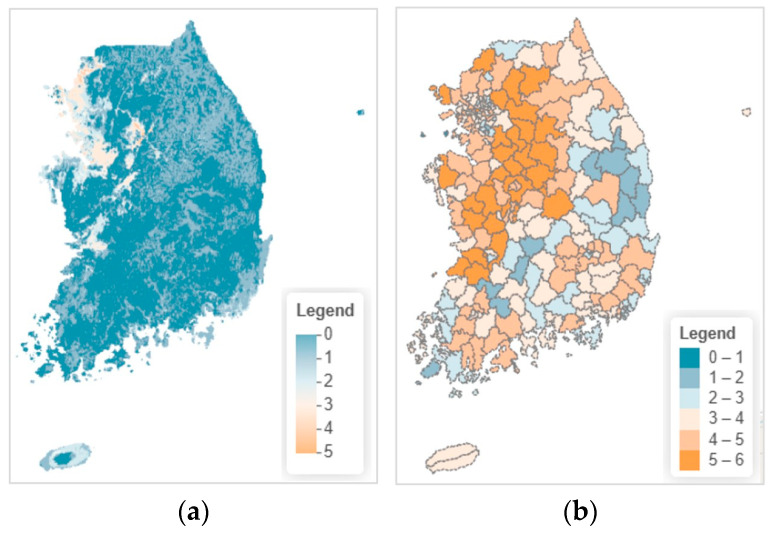
Current (2010) species richness patterns: (**a**) pixel-based distribution; (**b**) local government-level distribution.

Species-specific model performance, evaluated using the Random Forest model as a representative high-performing approach, revealed pronounced interspecific variation (Table 7, Figure 4). *S. chinensis* achieved exceptional predictive accuracy (mean ROC-AUC: 0.992 ± 0.003), with minimal variation across cross-validation iterations (range: 0.009). *D. suweonensis* (0.981 ± 0.004) and *H. yangi* (0.964 ± 0.014) similarly demonstrated excellent model performance. Moderate performance was observed for *P. chosenicus* (0.929 ± 0.021), *K. borealis* (0.918 ± 0.021), and *E. argus* (0.890 ± 0.041). Lower accuracy was observed for *M. reevesii* (0.784 ± 0.071) and *E. schrenckii* (0.715 ± 0.043). Standard deviation values were inversely correlated with mean ROC-AUC (Spearman’s ρ = −0.762, *p* = 0.028). Taxonomic comparison suggested higher model performance for amphibians (mean ROC-AUC: 0.948 ± 0.015) than for reptiles (0.845 ± 0.040; Mann–Whitney U test, *p* = 0.021), although this inference should be interpreted as exploratory given the small group sizes.

#### 3.1.4. Model Consensus by Species

Inter-model agreement, quantified through the coefficient of variation across 11 modeling approaches, is shown in Table 8. *S. chinensis* exhibited the highest consensus (CV = 5.2%). *D. suweonensis* (CV = 6.8%) and *H. yangi* (CV = 8.3%) also demonstrated high inter-model agreement. Moderate consensus was observed for *P. chosenicus* (CV = 10.5%), *K. borealis* (CV = 11.3%), and *E. argus* (CV = 13.6%). Low consensus was observed for *E. schrenckii* (CV = 18.7%) and *M. reevesii* (CV = 22.1%). A strong negative correlation was found between species-specific mean ROC-AUC and coefficient of variation (Spearman’s ρ = −0.905, *p* = 0.002).

### 3.2. Environmental Variable Importance

#### 3.2.1. Variable Contribution by Species

Across species, variable contributions differed markedly (Table 9). For *H*. *yangi*, precipitation of the driest month (BIO14; 0.443) and annual mean temperature (BIO1; 0.205) were the dominant predictors, followed by annual precipitation (BIO12; 0.159) and mean diurnal range (BIO2; 0.109). For *E*. *schrenckii*, BIO1 (0.330) was the primary predictor, with additional contributions from distance to water (0.111), slope (0.098), isothermality (BIO3; 0.098), and BIO14 (0.093). For *P*. *chosenicus*, elevation contributed most strongly (0.369), with secondary contributions from precipitation of the wettest month (BIO13; 0.120) and BIO12 (0.099). For *M*. *reevesii*, BIO13 (0.289), BIO1 (0.252), and distance to water (0.182) dominated. For *K*. *borealis*, slope (0.255) and distance to water (0.162) were highest, followed by BIO1 (0.120) and BIO13 (0.108). For *S*. *chinensis*, BIO14 (0.439) and BIO2 (0.392) explained most of the contribution profile. For *D*. *suweonensis*, BIO12 (0.283) and slope (0.244) were the leading predictors. For *E*. *argus*, elevation (0.205) and BIO14 (0.193) were most influential, followed by BIO2 (0.138) and BIO12 (0.138).

#### 3.2.2. Variable Importance Ranking

Taxonomic summaries indicated contrasting predictor emphasis between amphibians and reptiles (Table 10). Amphibians showed higher mean contributions from precipitation-related variables (BIO12–BIO14; precipitation sum = 0.402) and topographic variables (topographic sum = 0.290), whereas reptiles showed higher mean contributions from temperature-related variables (BIO1–BIO3; temperature sum = 0.398) and slightly higher hydrological dependence (hydrological sum = 0.096). Group comparisons were treated as exploratory given the small sample sizes (Table 10). Variable-importance rankings were consistent with contribution profiles and highlighted species-specific dominant predictors (Table 11), including BIO14 as the top-ranked predictor for *H*. *yangi* and *S*. *chinensis*, elevation for *P*. *chosenicus*, slope for *K*. *borealis* and *D*. *suweonensis*, and BIO13 and distance to water for *M*. *reevesii*.

#### 3.2.3. Taxonomic Patterns in Variable Contribution

Ranking Comparative analysis between amphibians and reptiles revealed pronounced taxonomic differences in environmental dependencies (Table 11). Reptiles exhibited significantly higher reliance on temperature-related variables (cumulative contribution: 0.398) compared to amphibians (0.230), representing a 1.7-fold difference (Welch’s *t*-test, t = 2.89, df = 4.2, *p* = 0.044). This pattern aligns with fundamental physiological differences between the two groups: reptiles, with keratinized skin and lower cutaneous water permeability, rely more heavily on behavioral thermoregulation and are thus more strongly constrained by thermal environments. In contrast, amphibians demonstrated significantly higher dependence on topographic variables (0.290 vs. 0.146 for reptiles; 2.0-fold difference; t = 3.45, df = 5.1, *p* = 0.018), particularly elevation and slope. This reflects amphibians’ greater sensitivity to fine-scale habitat structure, which influences microclimate, moisture retention, and breeding site availability. The higher contribution of slope for amphibians (mean: 0.152) compared to reptiles (0.046) is particularly notable, as many amphibian species require specific terrain features for breeding (e.g., gentle slopes for temporary pools in *K. borealis*, stream gradients for *H. yangi*).

Precipitation variables showed similar contributions between taxonomic groups (amphibians: 0.402; reptiles: 0.360; t = 0.67, df = 5.8, *p* = 0.530). However, the composition of precipitation importance differed: amphibians showed relatively balanced contributions across BIO12, BIO13, and BIO14, whereas reptiles exhibited more variable patterns, with *S. chinensis* showing extreme specialization on BIO14. Excluding *S. chinensis* as a statistical outlier (Grubb’s test, G = 2.87, *p* = 0.008), the revised reptilian mean for temperature variables decreased to 0.374, while precipitation variables decreased to 0.322. Hydrological variables (dist_water and wetness combined) showed marginally higher importance for reptiles (0.096) than amphibians (0.079), driven primarily by *M. reevesii* (dist_water contribution: 0.182) and *E. schrenckii* (0.111). The contribution of wetness was minimal across both groups (amphibians: 0.012; reptiles: 0.005).

#### 3.2.4. Variable Redundancy and Interaction Effects

Pearson correlation analysis of variable contributions across eight species revealed both expected redundancies and ecologically meaningful patterns (Table 12). Strong positive correlations were observed between BIO2 (mean diurnal range) and BIO3 (isothermality; r = 0.715, *p* = 0.047), reflecting their mathematical relationship. However, *S. chinensis* derived 45.7% of distributional variance from both variables (BIO2: 0.392, BIO3: 0.065), indicating that correlated predictors can provide complementary ecological information when species exhibit complex thermal requirements. BIO14 (precipitation of driest month) demonstrated notable independence from other precipitation variables (r = 0.231 with BIO12, r = 0.187 with BIO13), suggesting that dry-season moisture availability represents a distinct limiting factor not captured by total or wet-season precipitation metrics. Topographic and hydrological variables showed relatively weak intercorrelation. Elevation and slope exhibited modest correlation (r = 0.318), while topographic wetness index (wetness) showed weak correlations with all variables (|r| < 0.200). Despite this independence, wetness contributed minimally to species distributions (normalized contribution: 0.000–0.021), likely because 1 km resolution inadequately captures microhabitat moisture conditions relevant to herpetofaunal distributions. Distance to water showed moderate negative correlation with BIO14 (r = −0.412), though both variables represent conceptually distinct dimensions—spatial accessibility versus regional moisture availability. While BIO2 and BIO3 exhibited statistical correlation (r = 0.715), *S. chinensis* showed substantial contributions from both variables (BIO2: 0.392, BIO3: 0.065). The consistently low importance of wetness was observed across all species (normalized contribution: 0.000–0.021).

### 3.3. Environmental Niche Characteristics

#### 3.3.1. Niche Breadth Analysis

Shannon diversity index (H’) values ranged from 1.523 to 2.058, indicating substantial interspecific differences in environmental niche breadth (Table 13). Based on H’, species were classified as specialists (H’ < 1.60), moderate specialists (1.60 ≤ H’ ≤ 1.95), and generalists (H’ > 1.95). *Sibynophis chinensis* exhibited the narrowest niche (H’ = 1.523), with habitat suitability primarily associated with precipitation of the driest month (BIO14) and mean diurnal range (BIO2). *Hynobius yangi* (H’ = 1.628) and *Dryophytes suweonensis* (H’ = 1.753) also showed relatively narrow niches, with variable contributions concentrated on a small set of predictors, particularly dry-season or annual precipitation and topographic context. *Pelophylax chosenicus* and *Eremias argus* displayed intermediate niche breadths, with distributions most strongly related to elevation and dry-season precipitation in lowland settings. In contrast, *Elaphe schrenckii*, *Mauremys reevesii*, and *Kaloula borealis* showed broader niches (H’ ≥ 1.965) and more evenly distributed variable contributions, with temperature, precipitation, topography, and hydrological factors all contributing meaningfully. Overall, *S. chinensis*, *H. yangi*, and *D. suweonensis* were more specialist-like, exhibiting stronger dependence on a limited set of environmental drivers compared with the more generalist taxa.

#### 3.3.2. Species Similarity in Environmental Preferences

Hierarchical cluster analysis based on Random Forest variable contribution profiles grouped the eight species into three clusters (Table 14). The cophenetic correlation coefficient was 0.812. Cluster 1 contained *H. yangi* and *D. suweonensis*. In this cluster, precipitation variables, particularly precipitation of the driest month (BIO14) and annual precipitation (BIO12), together with topographic variables such as slope, showed the highest contributions to species distributions. Cluster 2 comprised *S. chinensis*, *E. argus*, and *P. chosenicus*. Species in this cluster showed relatively strong contributions from temperature variables (BIO1–BIO3) and elevation, in combination with precipitation of the driest month (BIO14). Cluster 3 included *E. schrenckii*, *M. reevesii*, and *K. borealis*. These species had more even contributions from climatic, topographic, and hydrological predictors, and they also showed the highest niche breadth values (H′ ≥ 1.965) reported in Table 13.

#### 3.3.3. Climate Vulnerability and Conservation Prioritization

Species were classified into climate-vulnerability tiers using transparent, a priori thresholds based on projected loss of suitable habitat area by the 2070s under SSP5-8.5, supported by niche breadth (H′) and model confidence (ROC-AUC) (Table 15). Projected habitat change (%) was calculated as the percent change in the number of suitable 1 km grid cells relative to the baseline climate, with positive values in Table 15 reported as the magnitude of habitat loss. We defined vulnerability levels as follows: High (habitat loss > 30%), Moderate (15–30%), and Low (<15%). Niche breadth and ROC-AUC were used to contextualize these tiers (e.g., narrow niches and high model confidence indicate higher sensitivity and stronger inference), but the tier assignment is anchored to the habitat-loss thresholds to avoid mixed criteria.

### 3.4. Species Richness Patterns Under Current and Future Climates

#### 3.4.1. Current Species Richness Distribution

Ensemble model projections revealed current (baseline: 2010) species richness ranging from 0 to 5 species per 1-km^2^ grid cell across South Korea (Figure 4a). High richness areas (4–5 species) were concentrated in western lowland plains, particularly Gyeonggi-do western region, Chungcheongnam-do west coast, and Jeollabuk-do western plains. These regions harbor overlapping habitats for lowland wetland-dependent species *(P. chosenicus*, *D. suweonensis*, *K. borealis*, *M. reevesii*), supported by extensive rice paddies, reservoirs, and wetland networks. Moderate richness (2–3 species) occurred widely across central inland and southern hilly regions, while low richness (0–1 species) characterized high-elevation areas (Gangwon-do eastern mountains), major urban centers (Seoul, Busan, Daegu), and portions of the east coast. Spatial analysis revealed a general west-to-east and south-to-north declining gradient in species richness, closely associated with elevation, temperature, and agricultural land density. At the administrative unit scale, local governments exhibited richness classes ranging from 0–1 to 5–6 species (Figure 4b). The highest richness class (5–6 species) was restricted to west coast municipalities (Ganghwa-gun, Seosan-si, Gimje-si, Gunsan-si), where diverse wetland types coexist. Moderate to high richness (3–5 species) occurred in agricultural landscape mosaics of Gyeonggi-do and Chungcheongnam-do, while low richness (<2 species) characterized mountainous Gangwon-do districts and urban cores.

#### 3.4.2. Projected Changes Under Climate Scenarios

**Figure 5:**
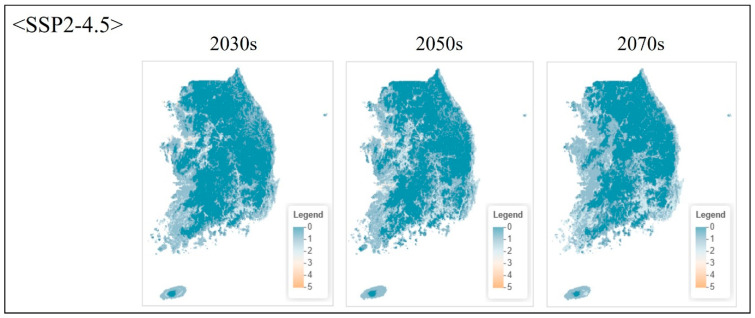
Projected species richness under moderate emission scenario (SSP2-4.5) at pixel level. Maps show ensemble model projections for three future periods.

Under the moderate emission scenario (SSP2-4.5), species richness remained relatively stable through the 2030s, with minor changes across most regions (Figure 5). By the 2050s, high-richness areas (cells containing 4–5 species) contracted by approximately 12–15% relative to current conditions, primarily in inland Chungcheongnam-do and eastern Jeollabuk-do. By the 2070s, high-richness area declined by 22%, accompanied by increased spatial fragmentation. Potential northward shifts in climatic suitability were also evident, with richness increasing in the southern Gangwon-do and northern Gyeongsangbuk-do mountain regions.

**Figure 6:**
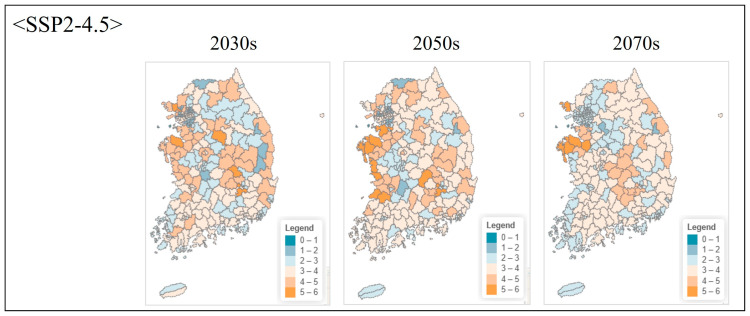
Projected species richness under moderate emission scenario (SSP2-4.5) at administrative unit level. Maps show local government-level aggregated species richness for three future periods.

**Figure 7:**
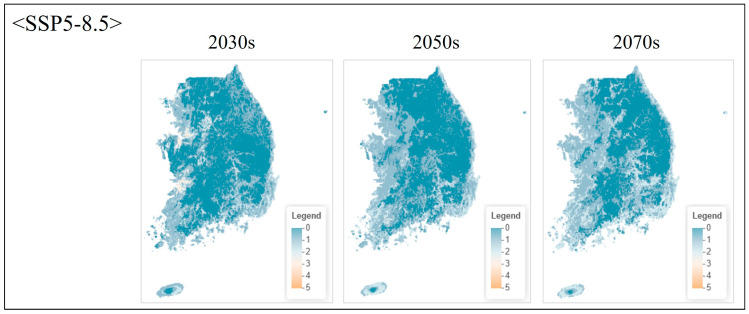
Projected species richness under high emission scenario (SSP5-8.5) at pixel level. Maps show ensemble model projections for three future periods.

**Figure 8:**
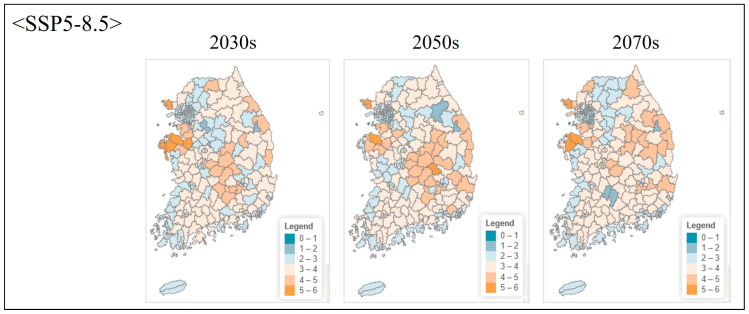
Projected species richness under high emission scenario (SSP5-8.5) at administrative unit level. Maps show local government-level aggregated species richness for three future periods.

The high emission scenario (SSP5-8.5) produced more rapid and severe changes (Figure 6). High-richness areas declined by 28% by the 2050s and 45% by the 2070s, corresponding to a reduction from approximately 7,240 km^2^ under current conditions to approximately 5213 km^2^ (2050s) and 3982 km^2^ (2070s), i.e., losses of ~2027 km^2^ and ~3258 km^2^, respectively. Spatial contraction was particularly pronounced in the western lowland plains, which currently support the highest diversity. By the 2070s, high-richness zones were restricted to limited areas in western Gyeonggi-do and northwestern Chungcheongnam-do, suggesting that these regions may function as critical but shrinking refugia under extreme climate trajectories (Figure 7 and Figure 8).

## 4. Discussion

### 4.1. Model Performance and Methodological Implications

Overall, the ensemble framework achieved strong predictive performance across species, supporting the usefulness of multi-algorithm SDMs for climate-impact assessment when single models may be sensitive to assumptions or sampling artefacts [15]. The higher performance of the AUC-weighted ensemble is consistent with the rationale that weighting can reduce the influence of weaker models while retaining complementary strengths from diverse algorithms [49,62]. Similar gains from ensemble approaches have been reported in SDM applications where algorithm performance varies across taxa and regions [63,64,65,66].

At the same time, performance metrics derived from random cross-validation may be optimistic when occurrence records are spatially clustered. Although we reduced clustering via 1 km thinning and partially addressed accessibility bias through weighting, residual spatial dependence may remain, especially for species with limited thinned sample sizes. Accordingly, model performance and projections should be interpreted as estimates of potential climatic suitability rather than deterministic predictions of realized occupancy.

### 4.2. Environmental Determinants of Species Distribution

#### 4.2.1. Taxonomic Differences in Environmental Requirements

The contrasting predictor patterns between amphibians and reptiles align with fundamental differences in hydroclimatic versus thermal constraints among ectotherms. Amphibians tended to show stronger dependence on precipitation-related variables, consistent with the requirement for suitable hydroperiods and moisture conditions for breeding and early development [3,12]. In contrast, reptiles showed greater influence of temperature-related predictors, reflecting the central role of thermal regimes in regulating activity time and physiological performance [5,6]. Similar taxonomic differences have been highlighted in other assessments of herpetofaunal responses to climate and environmental gradients [17,18,67,68].

#### 4.2.2. Species-Specific Ecological Implications

Species-level drivers further illustrate how climatic and landscape constraints interact with known habitat preferences. For *P*. *chosenicus* and *E*. *argus*, elevation emerged as a dominant driver, consistent with lowland distribution constraints and the role of elevation as a composite proxy for thermal regime and habitat context [10,69,70]. For *K*. *borealis*, slope contributed strongly, which may reflect breeding-site availability and moisture retention in low-slope settings where temporary pools can form and persist, while steep slopes promote rapid runoff and reduced soil moisture [71,72]. For *M*. *reevesii*, distance to water and wet-season precipitation (BIO13) were important, consistent with dependence on aquatic and riparian habitats and sensitivity to water availability [73]. These patterns underscore that projected climatic suitability shifts will interact with habitat structure and hydrological connectivity, especially in lowland landscapes where fragmentation can be pronounced.

### 4.3. Niche Breadth and Conservation Strategies

Niche breadth varied substantially among species, indicating different degrees of environmental specialization and potential sensitivity to climatic change. Species with narrow niche breadth are likely to be more vulnerable to shifts in limiting predictors and may require targeted, site-specific management and protection of remaining suitable habitat patches [21,22]. Conversely, broader-niche species may track changing suitability more readily, but their realized persistence will still depend on landscape connectivity and the availability of key habitat elements [74,75,76]. Therefore, conservation strategies should differentiate between specialist taxa requiring protection of specific environmental conditions and more generalist taxa that may benefit most from landscape-scale connectivity and habitat-network maintenance.

### 4.4. Species Associations and Community Dynamics

Clustering based on Spearman correlations of predictor contributions provides a concise summary of similarity in modeled limiting factors across species, which can be useful for generating hypotheses about shared environmental constraints [23,24]. However, such clustering does not directly represent ecological niche overlap, spatial co-occurrence, or species interactions, because it is derived from model-based contribution profiles rather than field-validated overlap or demographic processes [25]. Accordingly, cluster groupings should be interpreted as statistical similarity in modeled drivers and used as a guide for designing follow-up validation and coordinated management actions where appropriate [77,78].

### 4.5. Variable Redundancy and Model Optimization

Collinearity among climatic predictors can influence the stability of variable-importance attribution, particularly when correlated predictors convey overlapping information about temperature or precipitation regimes. Our correlation screening (|r| > 0.7) and the planned reporting of correlated predictor pairs support transparent interpretation of contribution patterns [37]. Previous SDM studies have emphasized that correlated predictors can shift relative contributions without necessarily altering predictive performance, and that ecological interpretation should therefore consider correlated-structure awareness rather than treating contributions as strictly causal effects [79,80]. Given the ecological relevance of each predictor to amphibian and reptile physiology and habitat constraints, retaining predictors while interpreting contributions cautiously is a defensible approach in ensemble SDM settings [81,82]. This is especially important when the goal includes species-specific ecological interpretation rather than purely predictive mapping [83,84].

### 4.6. Climate Change Vulnerability and Conservation Implications

#### 4.6.1. Species-Specific Vulnerabilities

Projected vulnerability differs among species because climatic constraints and habitat dependencies are not uniform. Amphibians dependent on stable hydroperiods and moisture availability may be sensitive to changes in dry-season precipitation and to increased variability that affects breeding success and recruitment [3,4]. Species with narrow niche breadth and concentrated dependence on a limited set of predictors may face higher risk under directional climate shifts, particularly where suitable habitats are already fragmented [21,22]. Conversely, taxa with broader niche breadth may show more diffuse climatic dependence, but can still be vulnerable when key habitat elements (e.g., wetlands, riparian corridors) are degraded or disconnected. These vulnerability patterns are consistent with broader herpetofaunal assessments linking climate stressors to physiological limits, altered phenology, and habitat-network constraints [6,85,86,87,88,89,90,91].

#### 4.6.2. Future Projections and Adaptive Conservation

Across scenarios, projections indicate redistribution and fragmentation of suitable habitats, with substantially stronger contraction under SSP5-8.5. Northward and upslope shifts are consistent with expected responses of ectotherms to warming, but the conservation value of emerging northern or montane suitability depends on whether species can reach and persist in these areas under real-world landscape constraints [92,93,94,95]. Similar studies have emphasized that climatically suitable refugia may not function as realized refuges without connectivity, appropriate microhabitats, and management to mitigate non-climatic stressors [96,97]. In lowland regions, losses in suitability and richness can be amplified by land-use pressures and wetland degradation, highlighting the need to maintain wetland–riparian networks and to protect remaining breeding habitats [98,99].

Effective adaptation should therefore combine (i) protection and management of current strongholds identified in suitability and richness maps, (ii) safeguarding prospective northern refugia, and (iii) improving connectivity to facilitate dispersal and range tracking where natural movement is limited [100,101,102]. Landscape-scale planning that prioritizes corridor integrity, riparian continuity, and wetland restoration is widely recognized as a core adaptation pathway for climate-sensitive taxa [103,104]. Where connectivity restoration is not feasible, site-scale interventions—such as maintaining hydroperiods during dry seasons, reducing barriers around breeding sites, and protecting lowland aquatic habitats—may reduce near-term vulnerability [105,106,107,108,109]. Importantly, projections represent potential climatic suitability and do not incorporate dispersal or adaptive capacity; therefore, management should interpret newly suitable areas as opportunity spaces that may require assisted connectivity or targeted interventions to become viable habitats [74,110]. Integrating SDM outputs with monitoring and adaptive management can improve decision-making under scenario uncertainty and support iterative conservation planning [104,111].

### 4.7. Study Limitations and Future Directions

Several limitations should be considered when applying these results. First, although thinning and weighting reduce spatial bias, heterogeneous survey effort may still influence modeled suitability patterns, especially for data-poor species. Second, 1 km resolution may not capture microhabitat features critical for breeding and thermoregulation, potentially underestimating the role of fine-scale wetlands or local refugia. Third, projections represent climatic suitability and do not explicitly incorporate dispersal constraints, demographic processes, biotic interactions, or adaptive capacity. Future work should therefore expand targeted surveys, validate projected refugia, integrate land-use and hydrological dynamics, and link suitability shifts to population-level risk assessment through demographic monitoring.

## 5. Conclusions

Using an ensemble SDM framework, we identified species-specific environmental drivers and projected climate-driven redistribution of suitable habitats for eight endangered amphibians and reptiles in South Korea. Predictor contributions indicated strong moisture sensitivity for several amphibians and pronounced thermal constraints for multiple reptiles, while niche breadth varied substantially among taxa. Richness projections revealed contraction and fragmentation of high-richness areas under both SSP scenarios, with localized northern gains that were insufficient to offset broader lowland losses, particularly under SSP5-8.5. Based on late-century habitat-loss thresholds supported by niche breadth and model confidence, we highlight a subset of species requiring the most urgent climate-adaptive attention. Conservation planning should combine protection of current strongholds with the safeguarding of prospective northern refugia and the enhancement of habitat connectivity to enable range tracking where natural dispersal is limited. Overall, our results provide a spatially explicit basis for prioritizing climate-adaptive conservation actions in South Korea.

## Figures and Tables

**Table 1:** Target amphibian and reptile species considered in this study, with national Red List categories and legal protection grades.

Class	Order	Scientific Name	Red List Category ^1^	ME Endangered Grade ^2^
Amphibia	Caudata	*Hynobius yangi*	EN	II
Amphibia	Anura	*Dryophytes suweonensis*	EN	I
Amphibia	Anura	*Pelophylax chosenicus*	VU	II
Amphibia	Anura	*Kaloula borealis*	VU	II
Reptilia	Squamata	*Sibynophis chinensis*	EN	I
Reptilia	Squamata	*Eremias argus*	VU	II
Reptilia	Squamata	*Elaphe schrenckii*	VU	II
Reptilia	Testudines	*Mauremys reevesii*	VU	II

^1^ EN: Endangered; VU: Vulnerable, based on the national Red List categories provided in the National Species List of Korea. ^2^ I, II: Endangered Wildlife Class I and II designated by the Korean Ministry of Environment.

**Table 2:** Number of occurrence records used for modeling before and after 1 km spatial thinning.

Species	Raw Occurrences (*n*)	After 1 km Thinning (*n*)
*H*. *yangi*	178	87
*E*. *schrenckii*	87	71
*P*. *chosenicus*	191	96
*M*. *reevesii*	37	25
*K*. *borealis*	356	122
*S*. *chinensis*	30	23
*D*. *suweonensis*	228	129
*E*. *argus*	45	23

Note: “After 1 km thinning” indicates the number of unique 1 km × 1 km grid cells containing occurrence records (one record retained per cell).

**Table 4:** Model performance evaluation metrics across all species and models.

Metric	Mean	Min	Max	Std
ROC-AUC	0.843	0.575	0.992	0.120
TSS	0.654	0.177	0.985	0.221
Kappa	0.460	0.047	0.797	0.231

Note: Values represent the average across all eight species and all single-algorithm species distribution models (Random Forest, GBM, GLM, FDA, MARS, ANN, MaxEnt, CTA, and SRE), excluding ensemble models. ROC-AUC = area under the receiver operating characteristic curve; TSS = true skill statistic; Kappa = Cohen’s kappa coefficient.

**Table 5:** Performance comparison of species distribution models across all species.

Model	Mean ROC-AUC	Min	Max	Std	Rank
EMW	0.897	0.715	0.992	0.099	1
EM	0.892	0.710	0.992	0.103	2
RF	0.889	0.660	0.992	0.108	3
GBM	0.885	0.679	0.988	0.105	4
GLM	0.864	0.682	0.977	0.118	5
FDA	0.860	0.670	0.988	0.125	6
MARS	0.844	0.642	0.978	0.134	7
ANN	0.830	0.638	0.975	0.135	8
MAXENT	0.816	0.682	0.913	0.071	9
CTA	0.806	0.575	0.987	0.137	10
SRE	0.690	0.587	0.773	0.074	11

Note: EMW = Ensemble Weighted Mean; EM = Ensemble Mean; RF = Random Forest; GBM = Gradient Boosting Machine; GLM = Generalized Linear Model; FDA = Flexible Discriminant Analysis; MARS = Multivariate Adaptive Regression Splines; ANN = Artificial Neural Network; MAXENT = Maximum Entropy; CTA = Classification Tree Analysis; SRE = Surface Range Envelope.

**Table 6:** Performance variability and stability indices across models.

Model	Mean ROC-AUC	Std	CV (%) *	Range	IQR ^†^	Stability Rank
MAXENT	0.816	0.071	8.7	0.231	0.089	1
SRE	0.690	0.074	10.7	0.186	0.098	2
EMW	0.897	0.099	11.0	0.277	0.124	3
EM	0.892	0.103	11.5	0.282	0.128	4
GBM	0.885	0.105	11.9	0.309	0.142	5
RF	0.889	0.108	12.1	0.332	0.156	6
GLM	0.864	0.118	13.7	0.295	0.167	7
FDA	0.860	0.125	14.5	0.318	0.189	8
MARS	0.844	0.134	15.9	0.336	0.198	9
ANN	0.830	0.135	16.3	0.337	0.201	10
CTA	0.806	0.137	17.0	0.412	0.223	11

* CV (Coefficient of Variation) = (Std/Mean) × 100. ^†^ IQR (Interquartile Range) = Q3–Q1. Note: Lower CV indicates more consistent performance across species.

**Table 7:** Species-specific model performance using Random Forest.

Species	Class	Mean ROC-AUC	Min	Max	Std	Range
*S. chinensis*	Reptilia	0.992	0.988	0.997	0.003	0.009
*D. suweonensis*	Amphibia	0.981	0.974	0.987	0.004	0.013
*H. yangi*	Amphibia	0.964	0.935	0.987	0.014	0.052
*P. chosenicus*	Amphibia	0.929	0.887	0.951	0.021	0.064
*K. borealis*	Amphibia	0.918	0.872	0.955	0.021	0.083
*E. argus*	Reptilia	0.890	0.805	0.938	0.041	0.133
*M. reevesii*	Reptilia	0.784	0.659	0.903	0.071	0.244
*E. schrenckii*	Reptilia	0.715	0.666	0.824	0.043	0.158
Amphibia Mean	-	0.948	-	-	0.015	
Reptilia Mean	-	0.845	-	-	0.04	
Overall Mean	-	0.897	-	-	0.027	

Note: Range = Max–Min. Values for taxonomic groups represent unweighted means.

**Table 8:** Species-specific model consensus across 11 modeling approaches.

Species	Mean ROC-AUC	Std	CV (%) *	Range (max-min)	Consensus Level
*S. chinensis*	0.912	0.047	5.2	0.219	Very High
*D. suweonensis*	0.894	0.061	6.8	0.272	High
*H. yangi*	0.878	0.073	8.3	0.305	High
*P. chosenicus*	0.851	0.089	10.5	0.286	Moderate
*K. borealis*	0.847	0.096	11.3	0.380	Moderate
*E. argus*	0.824	0.112	13.6	0.300	Moderate-Low
*E. schrenckii*	0.748	0.14	18.7	0.237	Low
*M. reevesii*	0.772	0.171	22.1	0.244	Very Low

* CV (Coefficient of Variation) = (Std/Mean) × 100. Lower CV indicates higher agreement among models. Consensus levels are based on CV: Very High (<7%), High (7–10%), Moderate (10–15%), Moderate-Low (15–20%), Low (20–25%), Very Low (>25%).

**Table 9:** Variable contribution of Random Forest model by species.

Species	BIO1	BIO2	BIO3	BIO12	BIO13	BIO14	Elevation	Slope	Dist_Water	Wetness
*H. yangi*	0.205	0.109	0.022	0.159	0.031	0.443	0.007	0.014	0.01	0.001
*E. schrenckii*	0.330	0.054	0.098	0.056	0.079	0.093	0.068	0.098	0.111	0.013
*P. chosenicus*	0.047	0.050	0.044	0.099	0.120	0.081	0.369	0.097	0.072	0.021
*M. reevesii*	0.252	0.041	0.08	0.035	0.289	0.034	0.080	0	0.182	0.006
*K. borealis*	0.120	0.041	0.098	0.056	0.108	0.088	0.061	0.255	0.162	0.012
*S. chinensis*	0.014	0.392	0.065	0.037	0	0.439	0.044	0.007	0.002	0
*D. suweonensis*	0.058	0.099	0.027	0.283	0.081	0.058	0.112	0.244	0.025	0.014
*E. argus*	0.064	0.138	0.065	0.138	0.048	0.193	0.205	0.008	0.069	0

Note: Values represent normalized proportional contribution (sum = 1.000 for each species). Variable importance was calculated using permutation importance in Random Forest. The highest contribution for each species is indicated by values > 0.200.

**Table 10:** Mean variable contribution by taxonomic group.

Variable	Amphibia Mean	Reptilia Mean	Reptilia *	Difference ^†^
BIO1	0.107	0.165	0.215	−0.058
BIO2	0.075	0.156	0.078	−0.081
BIO3	0.048	0.077	0.081	−0.029
BIO12	0.149	0.067	0.076	0.083
BIO13	0.085	0.104	0.139	−0.019
BIO14	0.168	0.190	0.107	−0.022
elevation	0.137	0.099	0.118	0.038
slope	0.152	0.046	0.059	0.106
dist_water	0.067	0.091	0.121	−0.024
wetness	0.012	0.005	0.006	0.007
Temperature sum ^‡^	0.230	0.398	0.374	−0.168
Precipitation sum ^§^	0.402	0.360	0.322	0.042
Topographic sum ^¶^	0.290	0.146	0.177	0.144
Hydrological sum **	0.079	0.096	0.127	−0.017

* Reptilia excluding *S. chinensis* (*n* = 3: *E. schrenckii*, *M. reevesii*, *E. argus*). ^†^ Difference = Amphibia Mean-Reptilia Mean (all four species). ^‡^ Temperature variables: BIO1, BIO2, BIO3. ^§^ Precipitation variables: BIO12, BIO13, BIO14. ^¶^ Topographic variables: elevation, slope. ** Hydrological variables: dist_water, wetness.

**Table 11:** Variable importance ranking by species (1 = highest importance, 10 = lowest).

Species	BIO1	BIO2	BIO3	BIO12	BIO13	BIO14	Elevation	Slope	Dist_water	Wetness
*H. yangi*	2	4	6	3	5	1	9	7	8	10
*E. schrenckii*	1	9	3	8	6	5	7	3	2	10
*P. chosenicus*	8	7	9	3	2	5	1	4	6	10
*M. reevesii*	2	6	4	7	1	8	4	10	3	9
*K. borealis*	3	9	5	8	4	6	7	1	2	10
*S. chinensis*	6	2	3	5	9	1	4	7	8	9
*D. suweonensis*	6	4	8	1	5	6	3	2	9	10
*E. argus*	8	3	7	3	9	2	1	5	6	10

**Table 12:** Pearson correlation matrix of variable contributions across eight species.

Variable	BIO1	BIO2	BIO3	BIO12	BIO13	BIO14	Elevation	Slope	Dist_Water	Wetness
BIO1	1	0.142	0.682	−0.245	−0.089	−0.312	−0.198	0.287	0.412	0.056
BIO2	0.142	1	0.715	0.089	−0.156	0.587	0.234	−0.098	−0.267	−0.189
BIO3	0.682	0.715	1	−0.123	−0.201	0.298	0.076	0.134	0.045	−0.112
BIO12	−0.245	0.089	−0.123	1	0.524	0.231	0.156	−0.089	−0.312	0.098
BIO13	−0.089	−0.156	−0.201	0.524	1	0.187	0.289	−0.167	−0.098	0.134
BIO14	−0.312	0.587	0.298	0.231	0.187	1	0.089	−0.234	−0.412	−0.156
elevation	−0.198	0.234	0.076	0.156	0.289	0.089	1	0.318	−0.156	0.023
slope	0.287	−0.098	0.134	−0.089	−0.167	−0.234	0.318	1	0.267	0.089
dist_water	0.412	−0.267	0.045	−0.312	−0.098	−0.412	−0.156	0.267	1	−0.045
wetness	0.056	−0.189	−0.112	0.098	0.134	−0.156	0.023	0.089	−0.045	1

Correlation significant at *p* < 0.10 level. Note: Correlations based on normalized variable contribution patterns across eight species. High correlation does not necessarily indicate ecological redundancy, as demonstrated by *S. chinensis*’ dual dependence on BIO2 and BIO3.

**Table 13:** Niche breadth and environmental specialization of eight endangered species.

Species	Shannon Index (H′)	Classification	Primary Variables (>15% Contribution)
*S. chinensis*	1.523	Specialist	BIO14 (43.9%), BIO2 (39.2%)
*H. yangi*	1.628	Moderate Specialist	BIO14 (44.3%), BIO1 (20.5%), BIO12 (15.9%)
*D. suweonensis*	1.753	Moderate Specialist	BIO12 (28.3%), slope (24.4%)
*P. chosenicus*	1.828	Moderate Specialist	elevation (36.9%)
*E. argus*	1.872	Moderate Specialist	elevation (20.5%), BIO14 (19.3%)
*K. borealis*	1.965	Generalist	slope (25.5%), dist_water (16.2%)
*M. reevesii*	2.011	Generalist	BIO13 (28.9%), BIO1 (25.2%), dist_water (18.2%)
*E. schrenckii*	2.058	Generalist	BIO1 (33.0%)

**Table 14:** Spearman rank correlation matrix of environmental variable contributions among species.

Species	*S.* *chinensis*	*H. yangi*	*D.* *suweonensis*	*P.* *chosenicus*	*K.* *borealis*	*E. argus*	*M.* *reevesii*	*E.* *schrenckii*
*S. chinensis*	1.000	0.524	0.429	0.738	0.310	0.714	0.238	0.190
*H. yangi*	0.524	1.000	0.905 **	0.595	0.429	0.476	0.333	0.357
*D. suweonensis*	0.429	0.905 **	1.000	0.619	0.548	0.500	0.405	0.452
*P. chosenicus*	0.738	0.595	0.619	1.000	0.595	0.810 **	0.524	0.500
*K. borealis*	0.310	0.429	0.548	0.595	1.000	0.524	0.786 **	0.738
*E. argus*	0.714	0.476	0.5	0.810 **	0.524	1.000	0.429	0.452
*M. reevesii*	0.238	0.333	0.405	0.524	0.786 **	0.429	1.000	0.595
*E. schrenckii*	0.190	0.357	0.452	0.500	0.738	0.452	0.595	1.000

** Correlation significant at *p* < 0.01. Note: Values represent Spearman’s rank correlation coefficients based on normalized variable contributions.

**Table 15:** Climate vulnerability assessment and conservation priorities.

Species	Niche Breadth (H′)	Model Performance (ROCAUC)	Projected Habitat Change *	Vulnerability Level	Conservation Priority
*S. chinensis*	1.523	0.992	42.30%	High	Immediate
*H. yangi*	1.628	0.964	38.70%	High	Immediate
*D. suweonensis*	1.753	0.981	31.50%	High	Immediate
*P. chosenicus*	1.828	0.929	24.80%	Moderate	High
*E. argus*	1.872	0.89	22.10%	Moderate	High
*K. borealis*	1.965	0.918	18.60%	Moderate	Moderate
*M. reevesii*	2.011	0.784	12.40%	Low	Moderate
*E. schrenckii*	2.058	0.715	8.90%	Low	Moderate

* Projected habitat change represents the percentage change in suitable habitat area compared to current conditions. Note: Vulnerability classification based on H′ < 1.80 (High), 1.80 ≤ H′ ≤ 1.95 (Moderate), H′ > 1.95 (Low).

## Data Availability

The occurrence records used in this study include sensitive localities for legally protected endangered species and are therefore not publicly available. Access may be granted for research purposes upon reasonable request, subject to institutional review and data-use conditions from the National Institute of Ecology and relevant agencies. Derived model outputs and summaries (e.g., variable-contribution tables and aggregated richness surfaces) are available from the corresponding author upon reasonable request.

## Abbreviations

The following abbreviations are used in this manuscript:

AUC	Area under the receiver operating characteristic curve
ROC	Receiver operating characteristic
TSS	True Skill Statistic
SDM	Species distribution model
